# Broad-Range Detection of Microorganisms Directly from Bronchoalveolar Lavage Specimens by PCR/Electrospray Ionization-Mass Spectrometry

**DOI:** 10.1371/journal.pone.0170033

**Published:** 2017-01-13

**Authors:** Måns Ullberg, Petra Lüthje, Paula Mölling, Kristoffer Strålin, Volkan Özenci

**Affiliations:** 1 Department of Clinical Microbiology, Karolinska University Hospital, Stockholm, Sweden; 2 Division of Clinical Microbiology, Department of Laboratory Medicine, Karolinska Institutet, Stockholm, Sweden; 3 Department of Laboratory Medicine, Faculty of Medicine and Health, Örebro University, Örebro, Sweden; 4 Department of Infectious Diseases, Karolinska University Hospital, Stockholm, Sweden; 5 Unit of Infectious Diseases, Department of Medicine Huddinge, Karolinska Institutet, Stockholm, Sweden; Wadsworth Center, UNITED STATES

## Abstract

The clinical demand on rapid microbiological diagnostic is constantly increasing. PCR coupled to electrospray ionization-mass spectrometry, PCR/ESI-MS, offers detection and identification of over 750 bacteria and *Candida* species directly from clinical specimens within 6 hours. In this study, we investigated the clinical performance of the IRIDICA BAC LRT Assay for detection of bacterial pathogens in 121 bronchoalveolar lavage (BAL) samples that were received consecutively at our bacterial laboratory for BAL culture. Commensal or pathogenic microorganisms were detected in 118/121 (98%) BAL samples by PCR/ESI-MS, while in 104/121 (86%) samples by routine culture (*P*<0.01). Detection of potentially pathogenic microorganisms by PCR/ESI-MS was evaluated in comparison with conventional culture-based or molecular methods. The agreement between positive findings was overall good. Most *Staphylococcus aureus*-positive PCR/ESI-MS results were confirmed by culture or species-specific PCR (27/33, 82%). The identity of *Streptococcus pneumoniae* could however be confirmed for only 6/17 (35%) PCR/ESI-MS-positive samples. Non-cultivable and fastidious pathogens, which were not covered by standard culture procedures were readily detected by PCR/ESI-MS, including *Legionella pneumophila*, *Bordetella pertussis*, *Norcadia* species and *Mycoplasma pneumoniae*. In conclusion, PCR/ESI-MS detected a broad range of potential pathogens with equal or superior sensitivity compared to conventional methods within few hours directly from BAL samples. This novel method might thus provide a relevant tool for diagnostics in critically ill patients.

## Introduction

Pneumonia and other infections of the lower respiratory tract (LRT) are serious health problems associated with high morbidity and mortality worldwide [1, 2]. Early optimal antimicrobial treatment is linked to improved patient outcome, reduced risk for resistance development and adverse side effects by the antimicrobials [3]. Although *Streptococcus pneumoniae* is the most common bacterial pathogen isolated from community-acquired pneumonia (CAP), a large proportion of CAP is caused by other pathogens, with considerable geographical variations [4, 5]. In contrast, Gram-negative species and *Staphylococcus aureus* dominate among bacteria isolated from hospital-acquired pneumonia (HAP) [6, 7]. The wide range of possible pathogens challenge the development of methods for rapid detection and identification, needed to ensure timely appropriate antimicrobial therapy.

Conventional culture-based investigation of LRT secretions is currently the method of choice for detection of bacterial pathogens in critically ill patients with suspected pneumonia [3, 8]. Culture-based approaches however often fail in detecting a causative agent. Prior antimicrobial therapy decreases the sensitivity of methods based on bacterial viability [9, 10], and certain pathogens are generally not cultivable with standard methods. In addition, these methods are time-consuming and labor-intensive. During the recent years, novel culture-independent techniques have been proposed as promising alternatives for detection and identification of pathogens from severe pneumonia. Different to samples from sterile body sites, commensal and colonizing microorganisms complicate the analysis in LRT secretions. While multiplex PCR assays have short turn-around times and are suited for detection of multiple pathogens simultaneously, the target panel is limited and information regarding commensal microorganisms is generally lacking. Diagnostic PCR-kits designed for sepsis diagnosis, e.g. LightCycler SeptiFast from Roche Diagnostics [11] have broader coverage but lack important respiratory pathogens. Similarly, poor performance and insufficient coverage has been reported for the Unyvero multiplex PCR device (Curetis AG) for direct point-of-care detection of respiratory pathogens [12, 13]. Assays such as the RB5 Anyplex II kit (Seegene) are designed for detection of few atypical bacterial pathogens only. There is thus an obvious need for a rapid method covering both common and atypical microorganisms directly from LRT specimens.

PCR/ESI-MS overcomes these limitations by combining multiple broad range PCR reactions with electrospray ionization-mass spectrometry (PCR/ESI-MS), which rapidly provides sequence information from the generated amplicons [14]. Subsequent bioinformatical analysis with an integrated database enables identification of species and resistance determinants. An internal calibrant molecule in each PCR reaction permits semi-quantitation of the detected genomes. The first PCR/ESI-MS version for clinical research, the Ibis T5000 universal biosensor platform (Ibis Biosciences, an Abbott company), used an assay kit for identification of over 600 bacteria and *Candida* species. Evaluation of this assay on brochoalveolar lavage (BAL) samples showed however suboptimal performance, with a concordance to standard culture-based methods of 66% for detection of bacterial pathogens [15]. Further development led to the PLEX-ID instrument (Abbott). The concordance rate of this analysis with conventional methods was 77% for detection of bacterial pathogens and 45% when microorganisms from the normal flora were included [16]. It should be noted that the applied assay was intended for use in sterile fluids and not BAL. A re-designed, research-use-only version of the instrument was recently tested in LRT secretions. This study demonstrated the improved detection of bacterial pathogens in patients under antibiotic treatment with 49/104 (47%) hits in culture-negative samples [17]. However, the study considered only primary detections for evaluation and performance on polymicrobial samples was neglected.

The lack of a gold standard method hampers evaluation of discrepant findings originated from conventional culture-dependent methods and PCR/ESI-MS. In the hitherto published studies, detections from PCR/ESI-MS were not systematically confirmed by complementing methods when culture results were missing. This situation makes it extremely difficult to interpret the quality of the PCR/ESI-MS results reported previously.

In November 2014, a PCR/ESI-MS platform was CE-marked and became commercially available with the product name IRIDICA. The aim of the present study was to evaluate its performance in detection of respiratory pathogens in clinical BAL samples, using the IRIDICA BAC LRT Assay (Ibis Biosciences, Abbott Molecular, Des Plaines, IL). Discrepant results for selected bacterial species were further analyzed with species-specific or broad range-PCR. As the IRIDCA BAC LRT Assay occasionally reported “Fungus no ID”, we also aimed to study if such result was linked to any positive fungal culture results.

## Materials and Methods

### Study material

All BAL samples included in this study (*n* = 121) were part of standard hospital care and taken as per routine practice. Median age of the patients was 60 years (range 0–90 years) and the majority was male (83/121, 69%). Most samples were collected at the clinic for pulmonary diseases/allergy (72/121, 60%), intensive care units, hematology or transplantation (29/121, 24%), or at the clinic for infectious diseases (12/121, 10%). Consecutive BAL samples sent to the Division of Clinical Microbiology, Karolinska University Laboratory Huddinge, Stockholm, Sweden, for standard microbiological diagnostic between May 2014 and March 2015 were subjected to routine culture-based diagnostic and the remaining samples was stored at -20°C for subsequent analysis by PCR/ESI-MS.

### IRIDICA PCR/ESI-MS

Bacterial and *Candida* DNA was detected and semi-quantified using the IRIDICA BAC LRT Assay (Abbott), designed to detect 780 bacterial and *Candida* species and four antibiotic resistance markers, *mecA*, *vanA*/*vanB* and *blaKPC*. For a defined panel of non-*Candida* fungal DNA, the assay reports “Fungus detected—No ID can be provided”. The analysis requires approximately six hours, and up to six samples including a negative control can be run simultaneously. From each sample, an aliquot of 100 μl BAL was analyzed. Samples were processed according to the manufacturer’s recommendation, including mechanical and chemical lysis; extraction of DNA followed by PCR, using 18 primer pairs in 16-well pre-coated PCR strips; desalting and purification of the amplicons; and analysis by ESI-MS. Species identification was achieved by bioinformatical analysis with an integrated software and database. Quantitation of detected genomes was based on internal calibrant molecules in each reaction and was reported as a semi-quantitative “level” for each detection.

### Lower detection limit for *Staphylococcus aureus* by PCR/ESI-MS

The methicillin-resistant *S*. *aureus* (MRSA) strain CCUG 31966 was used to establish the lower detection level PCR/ESI-MS using the IRIDICA BAC LRT Assay for this species as well as for the *mecA* resistance determinant. Bacterial colonies were suspended in PBS to a McFarland 0.5. A 10-fold dilution series was prepared in PBS and dilutions 10^−3^, 10^−4^, 10^−5^ and 10^−6^ were analyzed in 4–6 replicates. The dilution, for which both *S*. *aureus* and *mecA* was detected in all runs was regarded as limit for reliable detection (lower detection limit).

### Routine culture-based microbiological diagnostics

A quantitative culture technique was employed for routine culture-based analysis of the clinical samples. This included aerobic and anaerobic culture on non-selective agar (blood agar); a crystal violet-containing blood agar plate with an optochin disc at 5% CO_2_ for selection and identification of *S*. *pneumoniae*; a chocolate agar plate with an oleandomycin disc at 5% CO_2_ for selection and identification of *Haemophilus influenzae*; and a cysteine-lactose-electrolyte-deficient (CLED) agar plate for identification of Enterobacteriaceae. All agar plates were inoculated with 10 μl BAL each and were incubated for a total of 2 days at 37°C. The lower limit of detection was 100 CFU/ml, and growth of >10^4^ CFU/ml was regarded significant. Primary respiratory pathogens were reported regardless of quantity, while potential pathogens, colonizers or contaminants were reported when dominating and/or present in significant quantities. Relevant microorganisms were identified by MALDI-TOF MS (Bruker Daltonik) or the Vitek 2 system (bioMérieux). A confidence score ≥2.0 or a probability score >93% were considered reliable species identification by MALDI-TOF MS or Vitek 2, respectively, as recommended by the manufacturers. Complementing analyses were carried out when indicated. Antimicrobial susceptibility testing was performed by disc diffusion according to EUCAST standards (EUCAST, http://www.eucast.org/). For verification of results from PCR/ESI-MS, results from related patient samples were recorded if available, including results from routine fungal culture.

### Definition of normal respiratory flora

Following bacterial species were in general regarded as part of the normal respiratory flora: α-hemolytic streptococci, coagulase-negative staphylococci, *Enterococcus* species, *Neisseria* species, *Rothia* species, *Corynebacterium* species and *Candida* species. Growth of these species was usually summarized and reported as “normal respiratory flora” from routine microbiological diagnostics.

### Verification of detections and species identification

Selected samples were analyzed for the presence of *S*. *aureus* and *S*. *pneumonia* by PCR targeting the *nuc* or *lytA* gene, respectively. DNA was extracted from a 500-μl aliquot of BAL using the MagNA Pure LC DNA Isolation Kit III (Roche Diagnostics, Mannheim, Germany) following the manufacturer’s protocol. PCR for *lytA* was performed on a LightCycler 2.0 (Roche Diagnostics) as described [18]; PCR for *nuc* was performed according to a previously described protocol [19] but adopted to the Rotorgene Q system (Qiagen) using 0.3 μM of each primer. The presence of other pathogens was verified by standard diagnostic PCR assay targeting respiratory tract pathogens (RB5 Anyplex II, Seegene; detecting *Mycoplasma pneumoniae*, *Chlamydophila pneumoniae*, *Legionella pneumophila*, *Bordetella pertussis* and *Bordetella parapertussis*), or by 16S rRNA sequencing.

### Statistical analysis

Differences were evaluated by the Mann Whitney or Kruskal-Wallis test as appropriate, categorical results were compared using Fisher’s exact test. *P*-values below 0.05 were considered statistically significant.

### Ethics statement

This study was performed in accordance with the Declaration of Helsinki. An ethical permission was not required since the samples were anonymized an deidentified before being obtained by the authors.

## Results

### PCR/ESI-MS detects overall more microorganisms than routine culture

**Fig 1:**
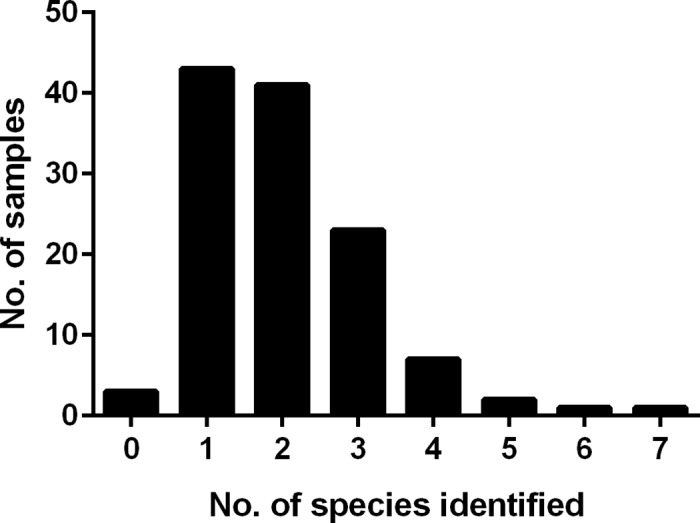
Detection of multiple microorganisms in bronchoalveolar lavage samples by PCR/ESI-MS. A total of 121 bronchoalveolar lavage samples were analyzed. The number of samples is depicted against the number of bacterial and *Candida* species identified per sample; various α-hemolytic streptococcal species are counted as one species.

A total of 245 microorganisms were detected by PCR/ESI-MS in 118/121 (98%) BAL samples included in the study (Fig 1). The microorganisms belonged to 60 different species, with 18 species regarded as primary or potential pathogens (referred to as pathogens hereafter, Table 1) for LRT infections. The remaining 42 species were considered part of the normal respiratory flora, colonizers or contaminants (Table 2). Pathogens were detected in 86/121 (71%) BAL samples, with two different pathogenic species in 15/86 (17%) samples. No detection was made 3/121 (2%) BAL samples. In contrast, “no growth” was reported for 17/121 (14%) samples after routine culture (*P*<0.01).

**Table 1:** Detection of bacterial pathogens in bronchoalveolar lavage by PCR/ESI-MS (IRIDICA).

Primary and potential pathogens	IRIDICA-positive	
	Total	Confirmed by culture and/or PCR
**Gram-positive bacteria**		
*Staphylococcus aureus*	33	27^a^
*Streptococcus pneumoniae*	17	6
*Corynebacterium pseudodiphteriticum*	1	-
**Gram-negative bacteria**		
*Haemophilus influenzae*	20	16
*Klebsiella pneumoniae*	4	4
*Enterobacter cloacae*-complex	5^b^	4
*Escherichia coli*	2^c^	2
*Escherichia vulneris*	1	-
*Proteus mirabilis*	2	1
*Proteus vulgaris*	1	-
*Klebsiella oxytoca*	1	-
*Pseudomonas aeruginosa*	8	7
*Stenotrophomonas maltophilia*	2	1
*Chryseobacterium indologenes*	1	-
**Atypical/ fastidious species**^**d**^		
*Nocardia* species	1	1
*Bordetella pertussis*	1	1
*Legionella pneumophila*	1	1
*Mycoplasma pneumoniae*	1	1

^a^Positive in culture or for *nuc*; one negative sample was not available for further investigation.

^b^For one sample, both *E*. *cloacae*-complex and *E*. *cancerogenus* was reported by IRIDICA; this result was counted as one isolate belonging to the *E*. *cloacae*-complex.

^c^For one sample, both *E*. *coli* and *E*. *coli/Shigella* was reported by IRIDICA; this result was counted as one *E*. *coli* isolate.

^d^Presence of these bacteria was confirmed by conventional PCR assays (*L*. *pneumophila*, *B*. *pertussis*, *M*. *pneumoniae*), sequencing (*Nocardia* species), special culture (*L*. *pneumophila*) or detection of urinary antigen (*L*. *pneumophila*).

**Table 2:** Detection of commensal flora in bronchoalveolar lavage by PCR/ESI-MS (IRIDICA).

Commensal microorganisms	Total
***Candida* species**	
*Candida albicans*	21
Other *Candida* species	7
**Gram-positive cocci**	
α-hemolytic streptococci	42
Coagulase-negative staphylococci	16
*Enterococcus* species	12
*Rothia* species	10
*Granulicatella adiacens*	8
*Gemella* species	3
**Gram-positive rods**	
*Corynebacterium* species	3
*Lysinibacillus sphaericus*	1
**Gram-negative cocci**	
*Neisseria* species	2
*Moraxella catarrhalis/nonliquefaciens*^a^	3
**Gram-negative rods**	
*Leclercia adecarboxylata*	1
*Actinobacillus capsulatus*	2
*Aggregatibacter segnis*	1
*Pseudomonas fluorescens*	1
**Anaerobe bacteria**	
*Lactobacillus* species	3
*Propionibacterium acnes*	2
*Fusobacterium nucleatum*	2
*Porphyromonas endodontalis*	1
*Veillonella* species	2

^a^In one of these samples, a *Moraxella* species was isolated by routine culture and identified as *Moraxella (Branhamella) catarrhalis* by MALDI-TOF MS.

### Performance of PCR/ESI-MS for detection of *Staphylococcus aureus*

**Fig 2:**
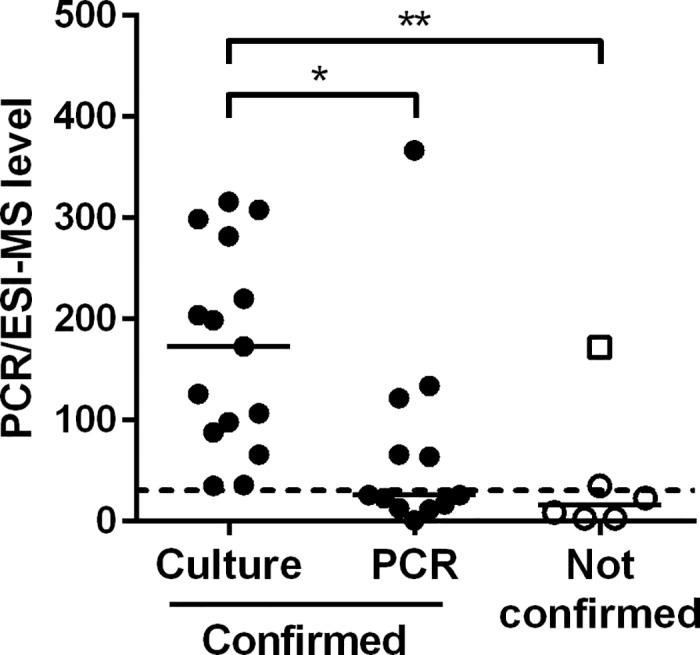
Semi-quantitative detection of *Staphylococcus aureus* by PCR/ESI-MS. *S*. *aureus* was detected in a total of 33 bronchoalveolar lavage samples. In 27 samples, the results were confirmed by culture (*n* = 15) or PCR for *nuc* (*n* = 12). Individual values with median are shown, comparison was made by Kruskal-Wallis with Dunn’s multiple comparison post-test; *P<0.05, **P<0.01. The dashed line indicates the mean level for the lower detection limit of *S*. *aureus* by PCR/ESI-MS (0.4×10^4^ CFU/ml), the open square indicates a sample not available for confirming tests.

*S*. *aureus* was among the most frequently detected potential pathogen, with 33 positive samples by PCR/ESI-MS. *S*. *aureus* was reported for 15/33 (45%) samples from routine culture, indicating dominating growth and significant numbers of colonies. Interestingly, semi-quantitative PCR/ESI-MS levels for *S*. *aureus*-specific DNA were significantly higher for these 15 specimens (Fig 2). Dose-response experiments with spiked samples defined a concentration of approximately 0.4×10^4^ CFU/ml as lower level for detection of this species, corresponding to PCR/ESI-MS levels of 35±6.9. All 15 samples with significant growth of *S*. *aureus* yielded levels above this threshold. In 12/18 (67%) culture-negative samples, detection of *S*. *aureus* by PCR/ESI-MS was confirmed by species-specific PCR. Five of the six remaining samples were tested negative by PCR; one was not available for further investigation. Together, the presence of *S*. *aureus* was verified by culture and/or PCR in 27/33 (82%) PCR/ESI-MS-positive samples. Among the studied material, there were no PCR/ESI-MS-negative samples with recorded growth of *S*. *aureus*.

### Performance of PCR/ESI-MS for detection of *streptococcus pneumoniae*

**Fig 3:**
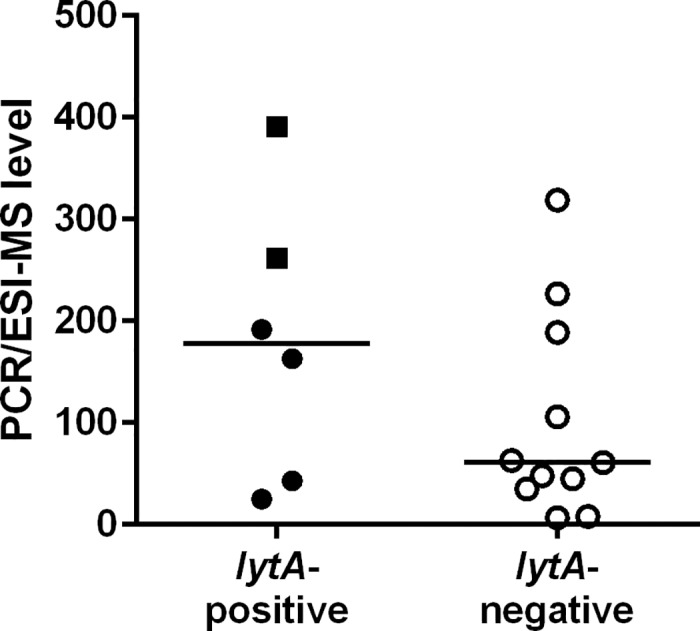
Detection of *Streptococcus pneumoniae* with PCR/ESI-MS. *S*. *pneumoniae* was detected in a total of 17 bronchoalveolar lavage samples. In 6 samples, the results were confirmed by PCR for *lytA*. Individual values with median are shown, the squares indicate culture-positive samples.

*S*. *pneumoniae* was detected in 17 BAL samples by PCR/ESI-MS. The presence of *S*. *pneumoniae* was however confirmed in only 6/17 (35%) samples, in two by positive culture and in another four samples by species-specific PCR (Fig 3). Semi-quantitative PCR/ESI-MS levels did not significantly differ between *lytA*-positive and *lytA*-negative samples (*P* = 0.35). High PCR/ESI-MS levels were however obtained for the two samples which were both *lytA*- and culture-positive. In addition, the C_T_-values for *lytA* indicated higher levels of the pathogen for culture-positive compared to culture-negative samples and overall, values correlated with semi-quantitative levels from the PCR/ESI-MS analysis (S1 Fig). Three culture-positive samples did not yield positive results for *S*. *pneumoniae* or any other streptococcal species by PCR/ESI-MS.

### Detection of *haemophilus influenza*

Detection of *H*. *influenzae* by PCR/ESI-MS was confirmed by culture in 16/20 (80%) BAL samples (Table 1). Semi-quantitative PCR/ESI-MS levels for 3/4 culture-negative samples were low or moderate (S2 Fig). Interestingly, two of these patients had been culture-positive for *H*. *influenzae* two weeks before the study sample was taken. Heavy growth of *Klebsiella pneumoniae* might have obscured the presence of *H*. *influenzae* in the fourth sample which yielded high PCR/ESI-MS levels (Table 3). Similarly, CFU levels were significantly higher in samples with positive result by PCR/ESI-MS, while sparse growth of *H*. *influenzae* was detected in three PCR/ESI-MS-negative samples (Table 3).

**Table 3:** Data for patients with discrepant results with respect to respiratory pathogens obtained by PCR/ESI-MS and routine culture-based analysis.

Patient	Culture report^a^ (CFU/ml)	PCR/ESI-MS (semi-quantitative levels)
Male, 36 yrs	***Haemophilus influenzae*** (10^2^−10^3^)	***Proteus mirabilis*** (127)
Female, 70 yrs	***Haemophilus influenzae*** (10^3^−10^4^), ***Streptococcus pneumoniae*** (10^3^−10^4^)	***Streptococcus pneumoniae*** (262)
Male, 35 yrs	***Haemophilus influenzae*** (10^2^−10^3^), Group G streptococcus (10^2^−10^3^)	Viridans/Salivarius Group *Streptococcus* (31), Viridans Group *Streptococcus* (18), *Gemella sanguinis* (11), Bacteria no ID (11)
Male, 84 yrs	Normal respiratory flora	***Haemophilus influenzae*** (47)^b^
Male, 74 yrs	No growth	***Haemophilus influenzae*** (37), *Candida albicans* (33)
Male, 41 yrs	Normal respiratory flora; **mold**	***Haemophilus influenzae*** (156)^b^, *Streptococcus* species (76), *Granulicatella adiacens* (52)
Male, 46 yrs	***Streptococcus pneumoniae*** (>10^4^), ***Klebsiella pneumoniae*** (>10^4^)	***Haemophilus influenzae*** (503)^c^, ***Klebsiella pneumoniae*** (14)
Male, 33 yrs	Normal respiratory flora	***Corynebacterium pseudodiphteriticum*** (120)
Male, 6 yrs	***Moraxella (Branhamella) catarrhalis*** (>10^4^), ***Streptococcus pneumoniae*** (10^3^−10^4^)	*Moraxella catarrhalis/nonliquefaciens* (547)^d^, *Streptococcus* species (49)
Male, 72 yrs	Yeast (10^2^−10^3^)	***Enterobacter cloacae*-complex** (169), *Granulicatella adiacens* (29), *Candida dubliniensis* (70), *Candida albicans* (109)
Female, 72 yrs	Normal respiratory flora^e^	***Escherichia vulneris*** (165), *Streptococcus pseudopneumoniae* (694), *Candida albicans* (302)
Female, 68 yrs	No growth	***Proteus vulgaris*** (461), *Staphylococcus lugdunensis* (5)
Male, 48 yrs	Normal respiratory flora	***Klebsiella oxytoca*** (11)^f^, ***Streptococcus pneumoniae*** (45), *Moraxella catarrhalis/nonliquefaciens* (32), *Candida albicans* (17)
Male, 56 yrs	***Streptococcus pneumoniae*** (10^2^−10^3^)	***Pseudomonas aeruginosa*** (237), Bacteria no ID (37)
Female, 70 yrs	No growth	***Stenotrophomonas maltophilia*** (304), *Candida albicans* (9)
Male, 29 yrs	Normal respiratory flora	***Streptococcus pneumoniae*** (43), ***Chryseobacterium indologenes*** (17), *Granulicatella adiacens* (77), Bacteria no ID (47)
Male, 49 yrs	***Klebsiella pneumoniae***	***Staphylococcus aureus*** (17)^g^, *Streptococcus* species (10), *Granulicatella adiacens* (90), Bacteria no ID (40)

^a^ Primary or potentially pathogenic species are indicated in bold; for results regarding *S*. *aureus* and *S*. *pneumoniae* see Fig 1 and Fig 2.

^b^ Positive culture for *H*. *influenzae* 2 weeks before study samples.

^c^ Positive culture for *H*. *influenzae* 2 weeks after study sample.

^d^ *Moraxella* species with comment “normal respiratory flora” from PCR/ESI-MS.

^e^ Positive fungal culture.

^f^ Positive culture for *Klebsiella oxytoca* in a sample taken 1 day before study sample.

^g^ *Staphylococcus aureus* in another respiratory sample taken the same day.

### PCR/ESI-MS allows detection of non-cultivable and fastidious pathogens

In four BAL samples, atypical pathogens were detected which were not covered by the routine culture-based method, namely *Legionella pneumophila*, *Bordetella pertussis*, *Mycoplasma pneumoniae* and *Nocardia* species. The detection of these microorganisms was confirmed by other methods in the identical or in related patient samples (Table 1).

### Resistance determinants *mecA*, *vanA* and *vanB* and *blaKPC*

The methicillin resistance-determinant *mecA* was detected in 21/121 (17%) BAL samples. Only 8/21 (38%) of these samples were also positive for *S*. *aureus*, indicating the potential presence of methicillin-resistant *S*. *aureus* (MRSA). Dose-response experiments showed that PCR/ESI-MS levels for *S*. *aureus* and the *mecA* gene were of similar range. An overall correlation between *mecA* and *S*. *aureus*-specific signals in these eight samples could however not been established, suggesting other origin of the *mecA* in at least part of the samples. Moreover, MRSA was not detected in any of the samples by culture, and significant growth of *S*. *aureus* was only reported for one sample. In one patient with high *mecA*-specific PCR/ESI-MS levels, however, a MRSA was isolated from another respiratory sample. Of the remaining *mecA*-positive samples, 12/13 displayed high PCR/ESI-MS levels (>100), and coagulase-negative staphylococci were detected in all of them, suggesting an associated of *mecA* to a none-*S*. *aureus* staphylococcal species.

There was no BAL sample that was *vanA*/*vanB*-positive. Similarly, no carbapenem-resistant or *blaKPC*-positive BAL sample was detected in the studied material.

### Report of fungal DNA without identification

While the BAC LRT Assay identifies *Candida* to genus level and eight *Candida* species furthermore to species level, a large panel of fungal DNA can be detected but not species-identified. The assay is moreover not designed for detection of fungi. However, for seven samples, the presence of fungal DNA was reported. Six of these samples were also sent for routine fungal diagnostics. In five of them, growth of fungi other than *Candida* was detected, including *Aspergillus fumigatus*, *Aspergillus versicolor*, *Penicillium* species, *Saccharomyces cerevisiae* and *Trichosporon ashii*.

## Discussion

PCR/ESI-MS is one of the most recent methods developed for detection and identification of microorganisms and selected resistance markers directly from clinical specimens. The aim of the present study was to evaluate the analytical performance of the PCR/ESI-MS platform IRIDICA in identification of microorganisms from BAL samples.

PCR/ESI-MS could identify 60 different microorganisms in 121 BAL samples and demonstrated an overall higher sensitivity compared to routine culture-based microbiological diagnostics, with identification of microorganisms in 15/17 (88%) culture-negative samples. In only one PCR/ESI-MS-negative sample, growth of normal respiratory flora was reported. Detailed microbiological diagnostics of BAL samples may be valuable information to the clinician depending on the clinical status of the patient.

When the analytical performance of PCR/ESI-MS in detection of primary or potential pathogens was analyzed, we found that the method in general was not inferior to culture-based methods in detection of these microorganisms. The vast majority of pathogens detected by culture, 52/60 (87%), could be detected by PCR/ESI-MS.

Identification of *S*. *pneumoniae* and differentiation from other α-hemolytic streptococci is notoriously difficult [20, 21]. Among the sample material investigated in this study, only 6/17 (35%) detections of *S*. *pneumoniae* were confirmed by culture or additional molecular analysis. In contrast, *S*. *pneumoniae* could not be identified in three samples with positive culture results. Interestingly, multiple matches were reported for the two samples with positive culture results, specified as *Streptococcus mitis/pneumoniae* and *S*. *pneumoniae*. This result was not displayed for any of the culture-negative samples. The present data indicates that PCR/ESI-MS has limited specificity and sensitivity in detection of *S*. *pneumoniae*. The low number of clinical isolates detected in BAL cultures does however not allow solid conclusions and further studies are needed. Based on the findings presented here, other tests complementary to PCR/ESI-MS are recommended to determine the presence or absence of *S*. *pneumoniae* if clinically indicated.

We experienced a similar problem in identification of *Moraxella catarrhalis*, which was reported as *Moraxella catarrhalis/nonliquefaciens* belonging to the normal flora. Problems to differentiate the two *Moraxella* species by molecular methods have previously been reported and been related to limited genomic sequence data for design of specific primers [13].

While all detected microorganisms are reported as positive finding by PCR/ESI-MS, culture reports include some degree of evaluation by a clinical microbiologist. Based on a low number of colonies or the presence of other fast-growing microorganisms, opportunistic or slow-growing pathogens might not be reported. This may explain the majority of the discrepancies observed for detection of *S*. *aureus*, a colonizer of the upper respiratory tract in up to 30% of adults [22, 23]. Samples with *S*. *aureus*-negative culture reports yielded overall significantly lower PCR/ESI-MS levels. Moreover, bacteria belonging to the normal flora were detected in 12/18 (67%) of these low-levels samples, but only in 4/15 (27%) samples, for which *S*. *aureus* was considered relevant by culture (*P*<0.05). These observations allow the suggestion that most discrepancies between culture reports and PCR/ESI-MS results are based on clinical interpretation of culture results rather than on false-positive reactions by PCR/ESI-MS. The present data also suggests that semi-quantitative levels and co-detection of commensal flora are relevant information for clinical interpretation of results from PCR/ESI-MS, in particular with respect to detection of *S*. *aureus*. PCR/ESI-MS results from BAL samples should thus be interpreted similarly to culture results. Microorganisms that are regarded as normal respiratory flora in BAL culture should reasonably be indicated as being part of the normal respiratory flora even when reporting results from PCR/ESI-MS on BAL.

In the present study, we were able to detect non-cultivable and fastidious pathogens with PCR/ESI-MS, i.e. *L*. *pneumophila*, *M*. *pneumonia*, *Nocardia* species and *B*. *pertussis*, which were overlooked by routine standard culture. Rapid detection of atypical pathogens from BAL samples by PCR/ESI-MS might be important in the clinical routine.

In addition, detection of fungal DNA by PCR/ESI-MS proved to be a reliable indication for the presence of non-*Candida* fungal species. Although no identification can be provided by the IRIDICA BAC LRT Assay, detection of fungal DNA in a sample may suggest that fungal-specific investigation is indicated and that antifungal treatment could be considered. It should however be noted that the IRIDICA BAC LRT Assay is not designed for detection of fungal species other than *Candida* and sensitivity for detection of molds by this assay has not been studied.

The IRIDCA BAC LRT Assay panel also includes selected major resistance determinants, i.e. *mecA*, *vanA*, *vanB*, and *blaKPC*. The only gene detected in the samples investigated here was *mecA*. In Sweden, only 1–3% of *S*. *aureus* from bloodstream infections are currently *mecA*-positive (http://ecdc.europa.eu) [24, 25]. Coagulase-negative staphylococci are however commonly detected as part of the normal flora or contaminant, and are frequently positive for *mecA*. In regions with relatively low prevalence of MRSA, the detection of *mecA* without physical linkage to a *S*. *aureus* isolate is thus of no diagnostic value in this kind of sample.

One of the limitations of the present study is the lack of clinical data. The clinical value of a comprehensive microbiological response to the clinicians could therefore not be assessed. In addition, the analysis was performed on consecutive BAL samples. In clinical practice however, samples from only a selected group of patients will be chosen for analysis by PCR/ESI-MS. The array of pathogens might thus probably differ and influence the overall performance of PCR/ESI-MS. A recent study on BAL samples from mechanically ventilated patients [26] indicated promising clinical performance of PCR/ESI-MS in comparison with other microbiological diagnostic approaches.

In conclusion, our study demonstrates overall reliable detection of respiratory pathogens by PCR/ESI-MS directly from BAL samples. PCR/ESI-MS thus provides an interestingly tool for the rapid detection of a wide range of pathogens from polymicrobial samples. This might be especially interesting for critically ill patients undergoing antimicrobial therapy at the time of sampling. Studies including clinical data are warranted to improve interpretation of the data.

## Supporting Information

S1 Fig (A) Culture results for *S*. *pneumoniae* in relation to C_T_-values for the *lytA* gene. (B) Relation between semi-quantitative PCR/ESI-MS levels and C_T_-values for the *lytA* gene.
(TIF)

S2 Fig Relation between PCR/ESI-MS results and CFU/ml (left) and between semi-quantitative PCR/ESI-MS levels and culture results for *H*. *influenzae*. In the sample indicated by a triangle, heavy growth of *K*. *pneumoniae* was detected.
(TIF)
