# Supplementary figures and images for: Broad-Range Detection of Microorganisms Directly from Bronchoalveolar Lavage Specimens by PCR/Electrospray Ionization-Mass Spectrometry
Source: PLoS One. 2017 Jan 13;12(1):e0170033. doi: 10.1371/journal.pone.0170033 (PMC5235381; doi:10.1371/journal.pone.0170033)

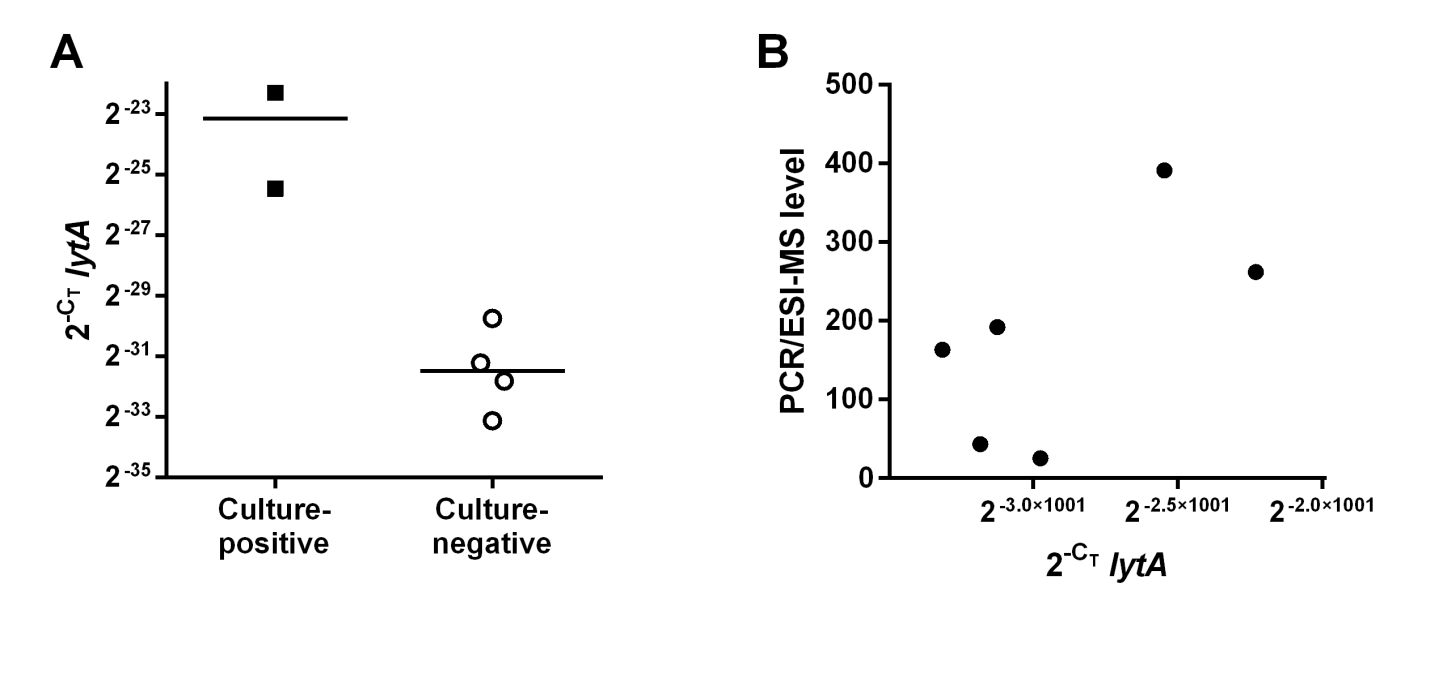

Supplement: S1 Fig — (A) Culture results for S. pneumoniae in relation to CT-values for the lytA gene. (B) Relation between semi-quantitative PCR/ESI-MS levels and CT-values for the lytA gene. (TIF) [file pone.0170033.s001.tif]

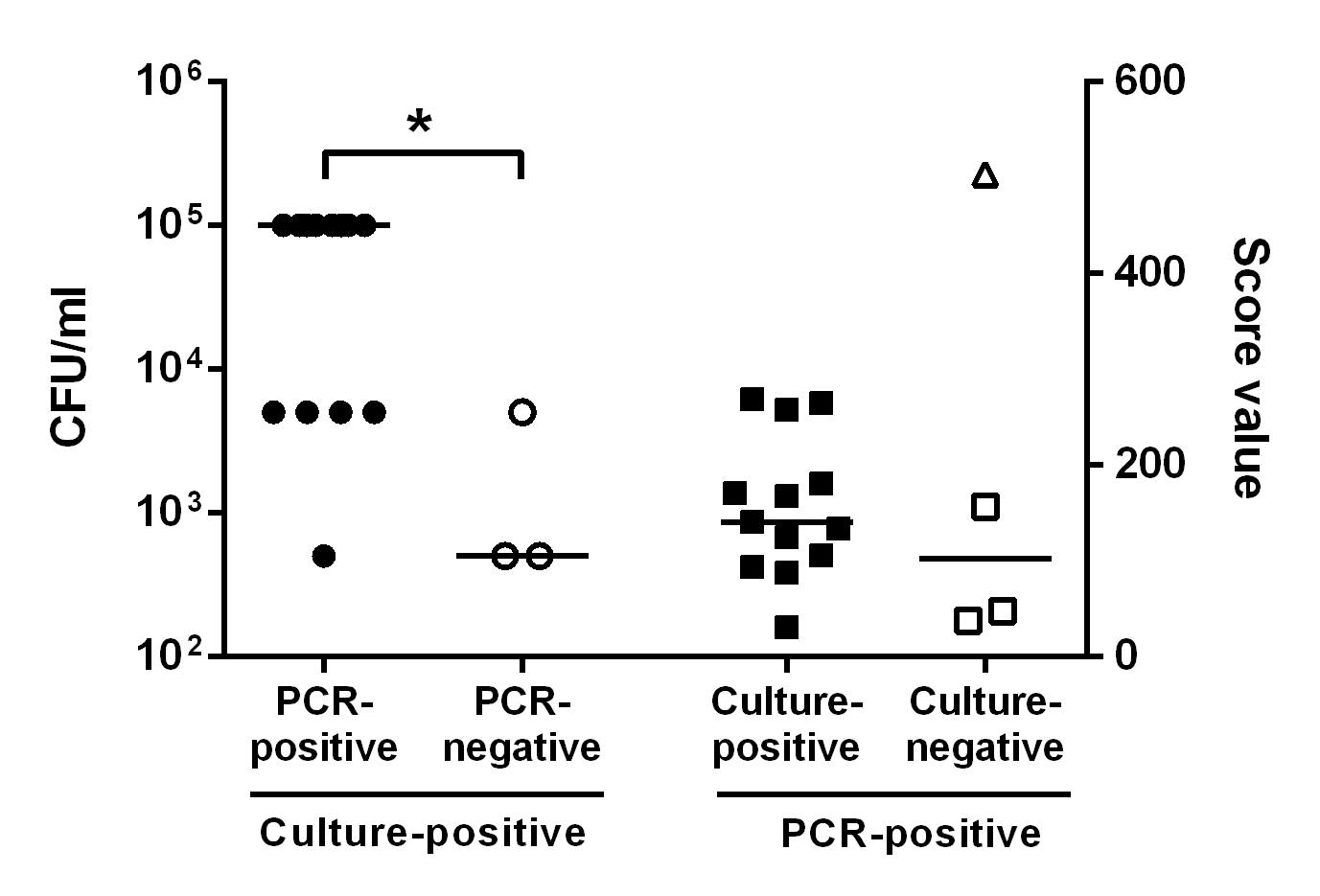

Supplement: S2 Fig — In the sample indicated by a triangle, heavy growth of K. pneumoniae was detected. (TIF) [file pone.0170033.s002.tif]
